# Timing of VV‐ECMO therapy implementation influences prognosis of COVID‐19 patients

**DOI:** 10.14814/phy2.14715

**Published:** 2021-02-01

**Authors:** Raphaël Giraud, David Legouis, Benjamin Assouline, Amandine De Charriere, Dumeng Decosterd, Marie‐Eve Brunner, Mallory Moret‐Bochatay, Thierry Fumeaux, Karim Bendjelid

**Affiliations:** ^1^ Intensive Care Unit Geneva University Hospitals Geneva Switzerland; ^2^ Faculty of Medicine University of Geneva Geneva Switzerland; ^3^ Geneva Hemodynamic Research Group Geneva Switzerland; ^4^ Laboratory of Nephrology Department of Medicine University Hospitals of Geneva Geneva Switzerland; ^5^ Department of Cell Physiology Faculty of Medicine University of Geneva Geneva Switzerland; ^6^ Intensive Care Unit, Réseau Hospitalier Neuchâtelois Site de Pourtalès Neuchatel Switzerland; ^7^ Intensive Care Unit Groupement Hospitalier de l'Ouest Lémanique Hôpital de zone de Nyon Nyon Switzerland

**Keywords:** ARDS, COVID‐19 pandemic, VV‐ECMO

## Abstract

**Introduction:**

Current knowledge on the use of extracorporeal membrane oxygenation (ECMO) in COVID‐19 remains limited to small series and registry data. In the present retrospective monocentric study, we report on our experience, our basic principles, and our results in establishing and managing ECMO in critically ill COVID‐19 patients.

**Methods:**

A cohort study was conducted in patients with severe acute respiratory distress syndrome (ARDS) related to COVID‐19 pneumonia admitted to the ICU of the Geneva University Hospitals and supported by VV‐ECMO from March 14 to May 31. The VV‐ECMO implementation criteria were defined according to an institutional algorithm validated by the local crisis unit and the Swiss Society of Intensive Care Medicine.

**Results:**

Out of 137 ARDS patients admitted to our ICU, 10 patients (age 57 ± 4 years, BMI 31.5 ± 5 kg/m^2^, and SAPS II score 56 ± 3) were put on VV‐ECMO. The mean duration of mechanical ventilation before ECMO and mean time under ECMO were 7 ± 3 days and 19 ± 11 days, respectively. The ICU and hospital length of stay were 26 ± 11 and 35 ± 10 days, respectively. The survival rate for patients on ECMO was 40%. The comparative analysis between survivors and non‐survivors highlighted that survivors had a significantly shorter mechanical ventilation duration before ECMO (4 ± 2 days vs. 9 ± 2 days, *p* = 0.01). All the patients who had more than 150 h of mechanical ventilation before the application of ECMO ultimately died.

**Conclusion:**

The present results suggest that VV‐ECMO can be safely utilized in appropriately selected COVID‐19 patients with refractory hypoxemia. The main information for clinicians is that late VV‐ECMO therapy (i.e., beyond the seventh day of mechanical ventilation) seems futile.

## Graphical abstract

Time‐course of COVID‐19 pneumoniafrom the onset of symptoms to the decision to implement VV‐ECMO according to the results of this study. HFNO, high‐flow nasal oxygen; CPAP, continuous positive airway pressure

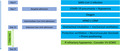

## 1 INTRODUCTION

The COVID‐19 pandemic has led to a significant number of hospitalizations for hypoxemic pneumonia, resulting in respiratory failure requiring orotracheal intubation and mechanical ventilation (Gattinoni, Chiumello & Rossi, 2020). In this regard, the most seriously affected patients presenting with acute respiratory distress syndrome (ARDS) may require veno‐venous extracorporeal membrane oxygenation (VV‐ECMO) as rescue therapy (Li et al., 2020). However, while the role of VV‐ECMO in severe ARDS has been clarified, its use in COVID‐19‐related ARDS is unclear, in particular due to the lack of knowledge and experience with SARS‐CoV‐2 pneumonia.

Different international organizations, including the Extracorporeal Life Support Organization (ELSO), have proposed recommendations for implementing ECMO in the context of COVID‐19 (Shekar et al., 2020). In Switzerland, under the aegis of the Swiss Society of Intensive Care Medicine, an algorithm detailing the indications and contraindications for setting up ECMO has been developed by our team and validated by all of the country's ECMO centers.

The canton of Geneva was one of the Swiss regions most affected by this pandemic (Primmaz et al., 2020). At our hospitals, more than 900 patients were hospitalized for COVID‐19 pneumonia, including 137 in our ICU with a diagnosis of ARDS.

In this retrospective study, we report on our experience, our basic principles, and our results in establishing and managing ECMO in critically ill COVID‐19 patients.

## 2 MATERIALS AND METHODS

An observational and retrospective study was conducted in patients with a diagnosis of COVID‐19 confirmed by a nasopharyngeal swab and/or bronchoalveolar lavage who were admitted to the Geneva University Hospitals ICU and who were supported by VV‐ECMO. The data were extracted from the institutional electronic medical record and the Patient Data Management System (PDMS) and Centricity critical care (Clinisoft®, GE Healthcare, General Electric Company). The collected data included patient characteristics, risk factors, and comorbidities before COVID‐19; SAPS II score on ICU admission; hemodynamic, respiratory, and biological parameters on admission and before ECMO setup; indication for continuous renal replacement therapy (CRRT); parameters and duration of ECMO support; ICU length of stay; and hospital length of stay. The local ethics committee approved the study and waved the informed consent (BASEC number: 2020‐00917).

**FIGURE 1:**
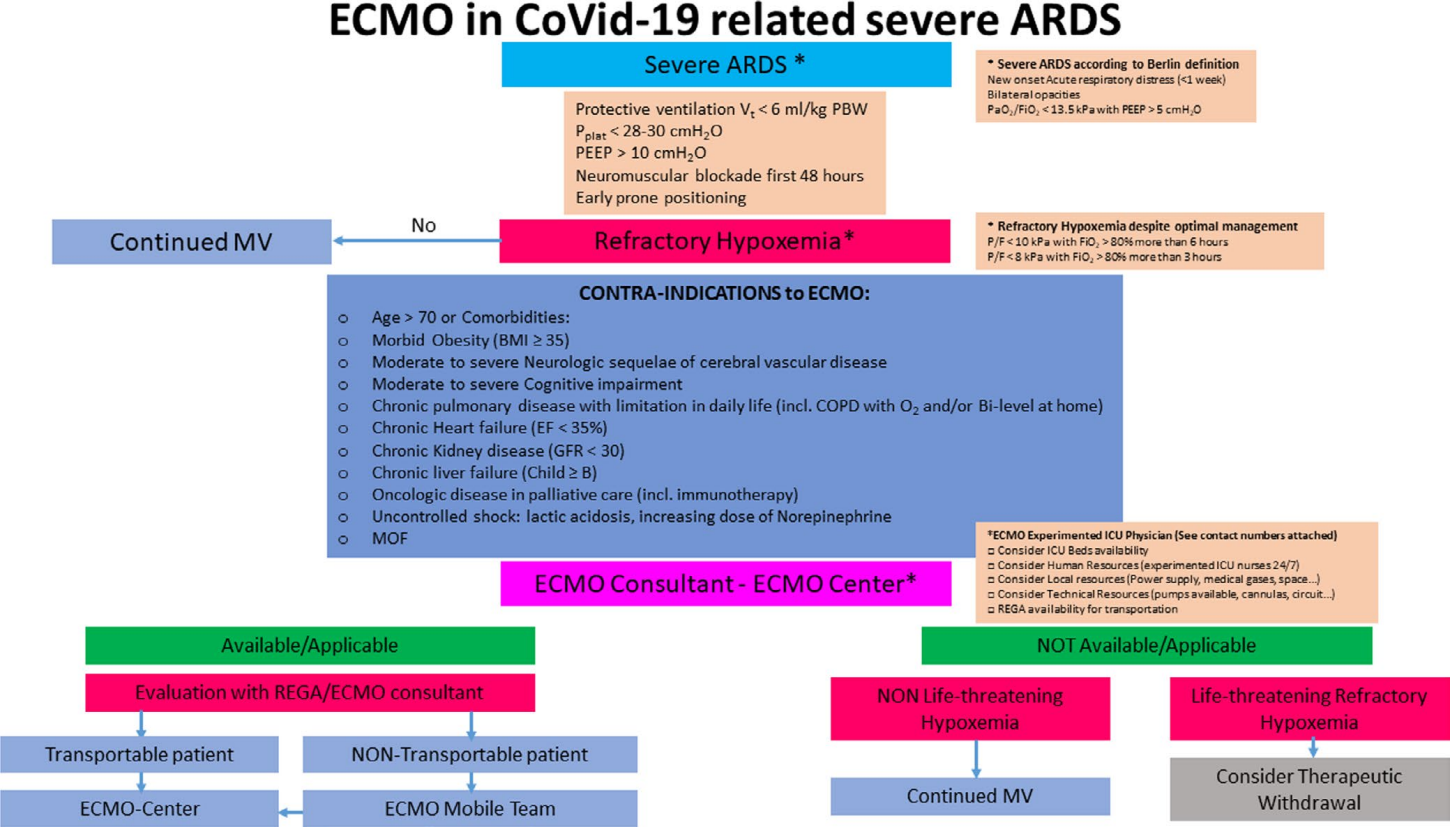
ECMO Guidelines for non‐ECMO centers in Switzerland. ARDS, Acute Respiratory Distress Syndrome; BMI, body mass index; COPD, chronic obstructive pulmonary disease; ECMO, extracorporeal membrane oxygenation; EF, ejection fraction; GFR, glomerular filtration rate; MOF, multiorgan failure; MV, mechanical ventilation; PEEP, positive end‐expiratory pressure; Pplat, plateau pressure; REGA, Swiss Air‐Rescue; Vt, tidal volume

This analysis includes all patients with COVID‐19 pneumonia initiated on VV‐ECMO from March 14, the date of admission of the first patient in intensive care requiring ECMO, to May 31, the date of discharge from the hospital of the last patient who received ECMO. The VV‐ECMO implementation criteria were defined according to an institutional algorithm validated by the local crisis unit and the Swiss Society of Intensive Care Medicine. This algorithm is presented in Figure 1. All cannulated patients presented with severe ARDS (as defined by the criteria of the Berlin definition) and hypoxemia refractory to optimal medical management. The decision to setup ECMO was made by the ECMO team of the Geneva University Hospitals (HUG). All the patients were cannulated percutaneously with echo‐guided punctures of the femorojugular vessels, while the placement of the guidewires and cannulas was carried out under transesophageal echocardiographic guidance.

Details concerning the geohealth situation of Switzerland (Geneva in particular), ECMO team and patient management under ECMO are presented in the electronic supplementary data.

### 2.1 Statistical analysis

Continuous variables are presented as the median and interquartile range or as the mean ± standard deviation, whereas categorical variables are expressed as percentages. Continuous variables were compared using the Mann–Whitney *U*‐test or Student's *t*‐test. Categorical variables were compared using the chi‐square test or Fisher's exact test. A value of *p* less than 0.05 was considered significant, and all p value tests were two‐tailed. All analyses were performed using GraphPad Prism 6 for Windows (GraphPad Software).

### 2.2 Machine learning

The dataset was first preprocessed. We generated a complete set of dummy variables from one or more factors. We further used the Yeo‐Johnson transformation on the continuous predictors and then centered and scaled them. Several machine learning algorithms were then used to build a predictive model of ICU death, including random forest (RF), L2 Regularized Linear Support Vector Machines with Class Weights (svmLinearWeights2), AdaBoost Classification Trees (adaboost), Support Vector Machines with Polynomial Kernel (svmPoly), Oblique Random Forest (ORFlog), Generalized Linear Model with Stepwise Feature Selection (glmStepAIC), and ROC‐Based Classifier (rocc). We used repeated cross‐validation with three separate 10‐fold cross‐validations as the resampling scheme. Accuracy was used to select the optimal model using the largest value. Variable importance evaluation was extracted for each model using the model's information. All measures of importance were scaled to have a maximum value of 100. These analyses were conducted using R software and Caret package (Kuhn, 2008).

## 3 RESULTS

Out of 137 ARDS patients admitted to our ICU, 10 patients were put under VV‐ECMO. All patients were treated with an empirical antiviral cocktail of hydroxychloroquine, azithromycin, and lopinavir +ritonavir before admission to the ICU. They were also all placed on empiric antibiotics (amoxicillin/clavulanic acid) during the first 5 days post‐intubation.

The age of the patients placed on ECMO was 57 ± 4 years. Their BMI was 31.5 ± 5 kg/m^2^. Their SAPS II score on admission was 56 ± 3. Four patients were treated chronically for high blood pressure, and four patients were treated chronically for diabetes. Two patients had controlled asthma. Clinical characteristics, comorbidities, and ECMO parameters are presented in Table 1.

**TABLE 1:** Clinical characteristics, comorbidities, ECMO parameters and clinical course of ECMO patients

Patients	1	2	3	4	5	6	7	8	9	10	All
Clinical characteristics											
Date of hospital admission	14.03.2020	21.03.2020	24.03.2020	31.03.2020	23.03.2020	24.03.2020	30.03.2020	28.03.2020	31.03.2020	05.04.2020	
Date of ICU admission	17.03.2020	21.03.2020	26.03.2020	31.03.2020	25.03.2020	24.03.2020	31.03.2020	01.04.2020	01.04.2020	05.04.2020	
Gender	F	F	F	F	M	M	M	M	F	M	5 M/5F
Age (years)	55	63	54	55	58	57	62	69	66	65	60 ± 5
Height (cm)	170	160	175	170	170	175	170	165	165	185	171 ± 7
Weight	73	90	80	85	59	75	70	63	70	95	76 ± 12
BMI (kg)	25	34	29	35	22	26	24	23	25	28	27.1 ± 4.4
SAPS II	53	58	53	59	52	75	52	48	65	67	58 ± 8
Date of mechanical ventilation	17.03.2020	21.03.2020	26.03.2020	31.03.2020	25.03.2020	24.03.2020	31.03.2020	01.04.2020	01.04.2020	08.04.2020	
Comorbidities											
Hypertension		Yes				Yes	Yes	Yes			4 (40%)
Diabetes			Yes			Yes	Yes				3 (30%)
Cardiovascular disease											0 (0%)
Malignancy											0 (0%)
Cerebrovascular disease							Yes				1 (10%)
Asthma/COPD	Yes				Yes						2 (20%)
Chronic kidney disease											0 (0%)
Murray score	3.3	3.3	3.5	3.3	3.5	3.8	3.3	3.3	3.5	3.5	3.4 ± 0.2
SAS	1	1	1	1	1	1	1	1	1	1	1
ECMO parameters											
P/F before ECMO (kPa)	8	8	12	10	7	12	10	11	6	12	9.6 ± 2.2
Lactate (mmol/L)	0.9	1.6	1.5	1.1	2.3	1.3	2.1	1.3	5	0.8	1.8 ± 1.2
Duration of mechanical ventilation before ECMO (Days)	5	1	6	3	7	8	8	8	11	11	7 ± 3
Perfusion Cannula (French)	19	19	19	19	21	21	21	21	19	21	20 ± 1
Drainage Cannula (French)	25	25	23	25	28	28	28	28	25	28	26.3 ± 2
AntiXa before ECMO (UI/ml)	0.21	0.25	0.34	0.44	0.24	0.25	0.29	0.24	0.34	0.32	0.29 ± 0.07
AntiXa under ECMO (UI/ml)	0.28	0.28	0.33	0.34	0.27	0.26	0.31	0.28	0.32	0.3	0.3 ± 0.03
Time on ECMO (Days)	5	12	15	9	11	43	25	28	15	25	19 ± 11
Mode of ECMO	VV	VV	VV	VV	VV	VV	VV	VV	VV	VV	VV (100%)
ICU length of stay (days)	12	15	26	16	13	45	32	37	30	33	25.9 ± 11.4
Outcome	Alive	Alive	Alive	Alive	Dead	Dead	Dead	Dead	Dead	Dead	4 Alive/6 Dead
Hospital length of stay (days)	22	30	42	45	15	45	33	41	30	44	34.7 ± 10.5
Adverse event					Intracranial hemorrhage						1 (10%)

Abbreviations: COPD, chronic obstructive pulmonary disease; ECMO, extracorporeal membrane oxygenation; ICU, Intensive Care Unit; P/F, PaO_2_/FiO_2_ ratio; SAS, Sedation Agitation Score.

**FIGURE 2:**
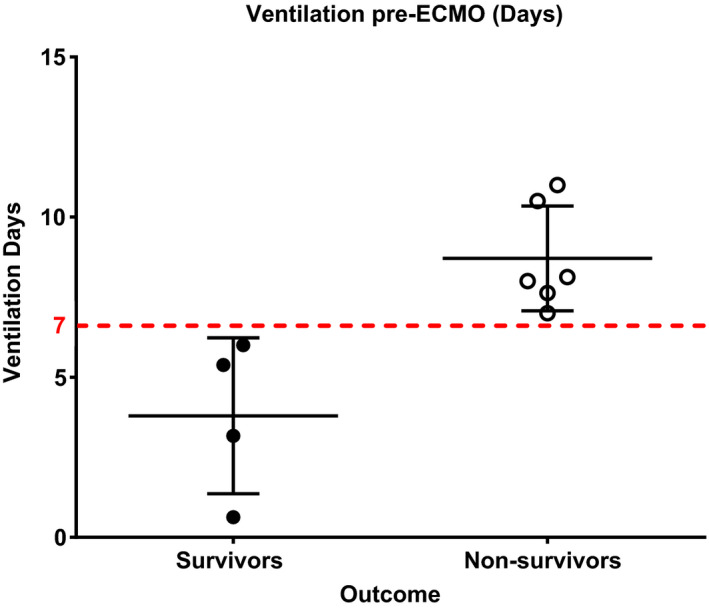
Scatter dot plot including mean and standard deviation of the number of pre‐ECMO ventilation time differences between survivors and non‐survivors

**FIGURE 3:**
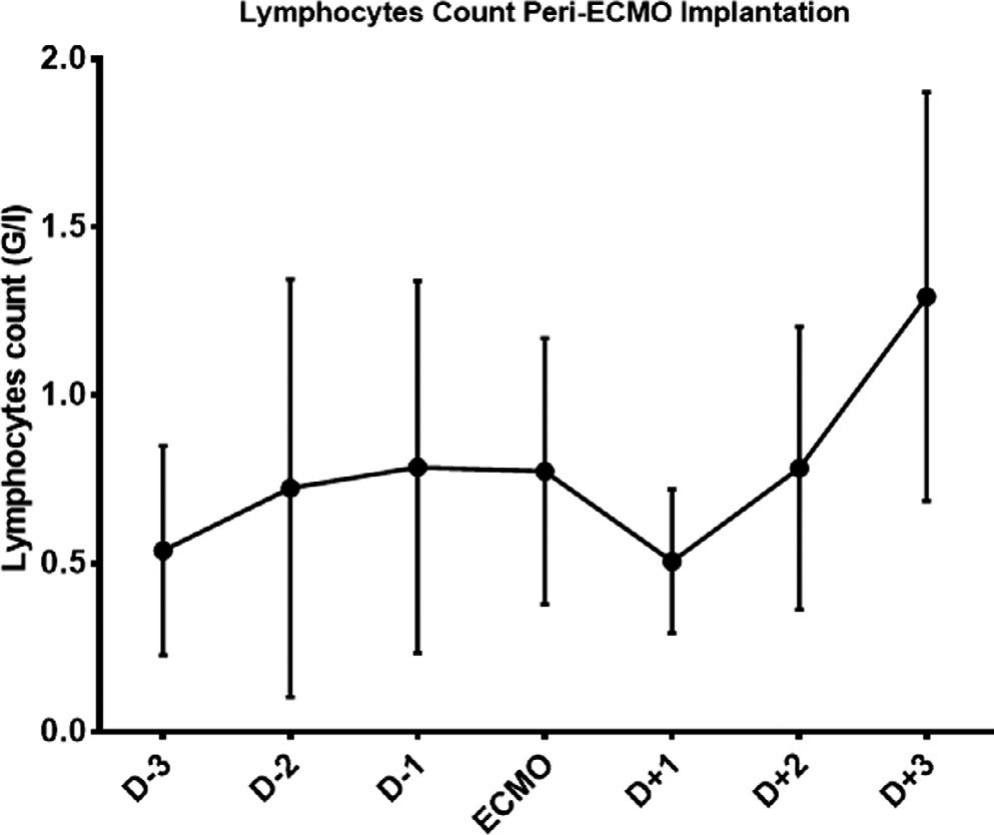
Lymphocytes count variation from 3 days before ECMO implantation to 3 days after ECMO implantation with mean and standard deviation

The hemodynamic, respiratory, and biological parameters are presented in Table 2. All patients had a high CRP value (184 ± 106 mg/l), with a low procalcitonin level (0.59 ± 0.38 µg/l) and lymphopenia (0.51 ± 0.31 G/l) and a white blood cell/lymphocyte ratio of 20 ± 21. The evolution of the number of lymphocytes around the initiation of ECMO is presented in Figure 2. There was a significant drop in the number of lymphocytes the day after starting ECMO. However, the lymphocyte count rose rapidly to return to preimplantation values by day 2 (Figure 3). No patient presented with renal failure needing extrarenal purification.

**TABLE 2:** Demographic and biological parameters of the patients implanted with VV‐ECMO at ICU admission and Pre‐ECMO. NS (non‐significant) indicates no significant statistical difference between parameters

	ICU admission	Pre‐ECMO	*p*
Hb (g/l)	126 ± 38	109 ± 29	NS
WCC (G/l)	7.8 ± 3.5	5.8 ± 1.4	NS
Lymphocytes (G/l)	0.61 ± 0.38	0.51 ± 0.31	NS
N/L ratio	15 ± 7	20 ± 21	NS
Thrombocytes (G/l)	247 ± 159	269 ± 101	NS
D‐dimers (ng/ml)	2782 ± 3122	2787 ± 1826	NS
CRP (mg/l)	161 ± 85	184 ± 106	NS
Procalcitonine (µg/l)	0.64 ± 0.42	0.59 ± 0.38	NS
Us‐Troponine (ng/l)	39 ± 27	42 ± 29	NS
Creatinine (µmol/l)	79 ± 22	68 ± 13	NS
Urea (mmol/l)	6.3 ± 3.4	7.6 ± 1.6	NS
Lactate (mmol/l)	1.2 ± 0.6	1.3 ± 0.3	NS
Temperature (°C)	37.6 ± 0.7	36.8 ± 0.8	NS

Abbreviations: BMI, body mass index; CRP, C‐reactive protein; CVVHDF, continuous veno‐venous hemodiafiltration; Hb, hemoglobin; N/L, neutrophils/lymphocytes ratio; P/F, PaO_2_/FiO_2_ ratio; PBW, predicted body weight; Us‐Troponin, ultrasensible troponin; Vt, tidal volume; WCC, white cells count.

Hemodynamically, all patients were stabilized with small doses of catecholamines (norepinephrine 0.04 ± 0.04 µg/kg per min), without circulatory failure. Patients were ventilated in controlled volume with protective ventilation (Vt at 6 ± 1 ml of PBW) with an optimized PEEP level (11 ± 2 cmH_2_O), paralyzed for the first 48 h. All patients had low compliance (19 ± 4 ml/cmH_2_O) and were severely hypoxemic with a P/F ratio at 10 ± 2 kPa. The Murray score was 3.4 ± 0.2. The number of prone positioning sessions was 2 ± 1, and all patients received iNo at an average mean rate of 13 ± 4 ppm. The comparison of demographic characteristics and biological parameters between admission to ICU and before ECMO did not show any significant difference (Table 2). The comparison of the ventilatory parameters between the postintubation and pre‐ECMO times showed a significant drop in tidal volume (420 ± 35 ml vs. 368 ± 41 ml, *p* = 0.0137), in the Vt/PBW ratio (6.4 ± 0.8 ml/kg vs. 5.7 ± 0.8 ml/kg, *p* = 0.0137), P/F ratio (34 ± 6 kPa vs. 10 ± 2 kPa, *p* = 0.002), and compliance (33 ± 4 ml/cmH_2_O vs. 19 ± 4 ml/cmH_2_O, *p* = 0.002), while the plateau pressure had significantly increased (23 ± 1 ml vs. 29 ± 2 ml, *p* = 0.002), reflecting respiratory worsening between intubation and the use of ECMO (Table 3).

**TABLE 3:** Ventilatory parameters post‐intubation and pre‐ECMO. NS (non‐significant) indicates no significant statistical difference between parameters

Ventilatory parameters	Post‐Intubation	Pre‐ECMO	*p*
Ventilatory mode	ACV	ACV	NS
Vt (ml)	420 ± 35	368 ± 41	0.0137
Vt/PBW (ml/kg)	6.4 ± 0.8	5.7 ± 0.8	0.0137
Pplat (cmH_2_O)	23 ± 1	29 ± 2	0.002
PEEP (cmH_2_O)	11 ± 1	11 ± 1	NS
RR (/min)	22 ± 4	21 ± 2	NS
P/F (kPa)	24 ± 6	10 ± 2	0.002
Crs (ml/cmH_2_O)	33 ± 4	19 ± 4	0.002

ACV, assist controlled ventilation; Crs, compliance of respiratory system; P/F, PaO_2_/FiO_2_ ratio; PEEP, positive end‐expiratory pressure; Pplat, plateau pressure; RR, respiratory rate; Vt, tidal volume.

**FIGURE 4:**
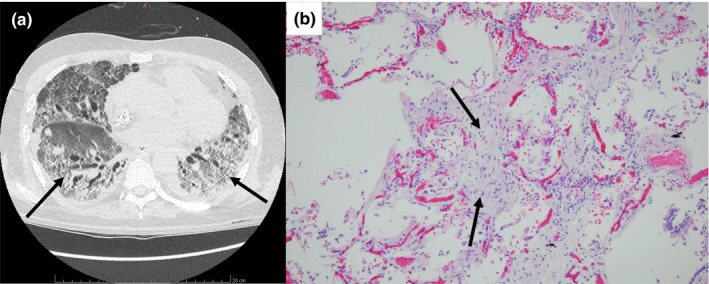
(a) CT‐Scan image and (b) anatomo‐pathological section of an extensive pulmonary fibrosis in patient 10. Arrows (a) Lung fibrosis and (b) intra‐alveolar fibrotic tissue, hematoxylin–eosin stain, Original magnification: ×100

The mechanical ventilation time before ECMO was 161 ± 76 h, the time under ECMO was 451 ± 270 h, the ICU length of stay was 26 ± 11 days, and the hospital length of stay was 35 ± 10 days. The mortality rate for patients on ECMO was 60%. Six patients died in this cohort, including one patient following a massive cerebral hemorrhage without neurosurgical sanction. The other five deaths occurred after a therapeutic withdrawal in the context of extensive pulmonary fibrosis (Figure 4), with no possibility of withdrawal either from the ECMO or from mechanical ventilation.

**FIGURE 5:**
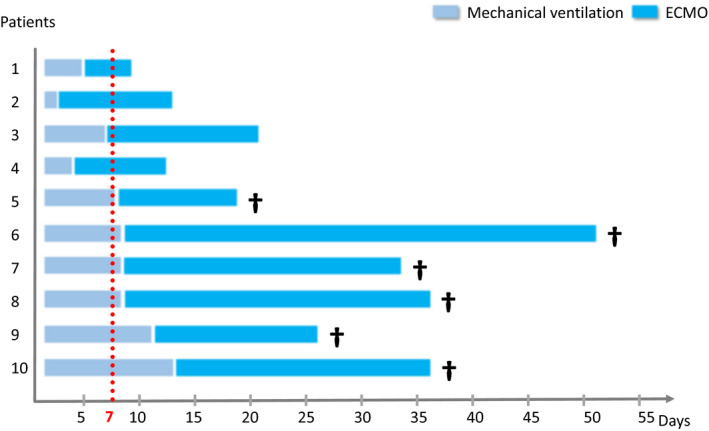
Time line of 10 ECMO patients with mechanical ventilation and ECMO durations

**FIGURE 6:**
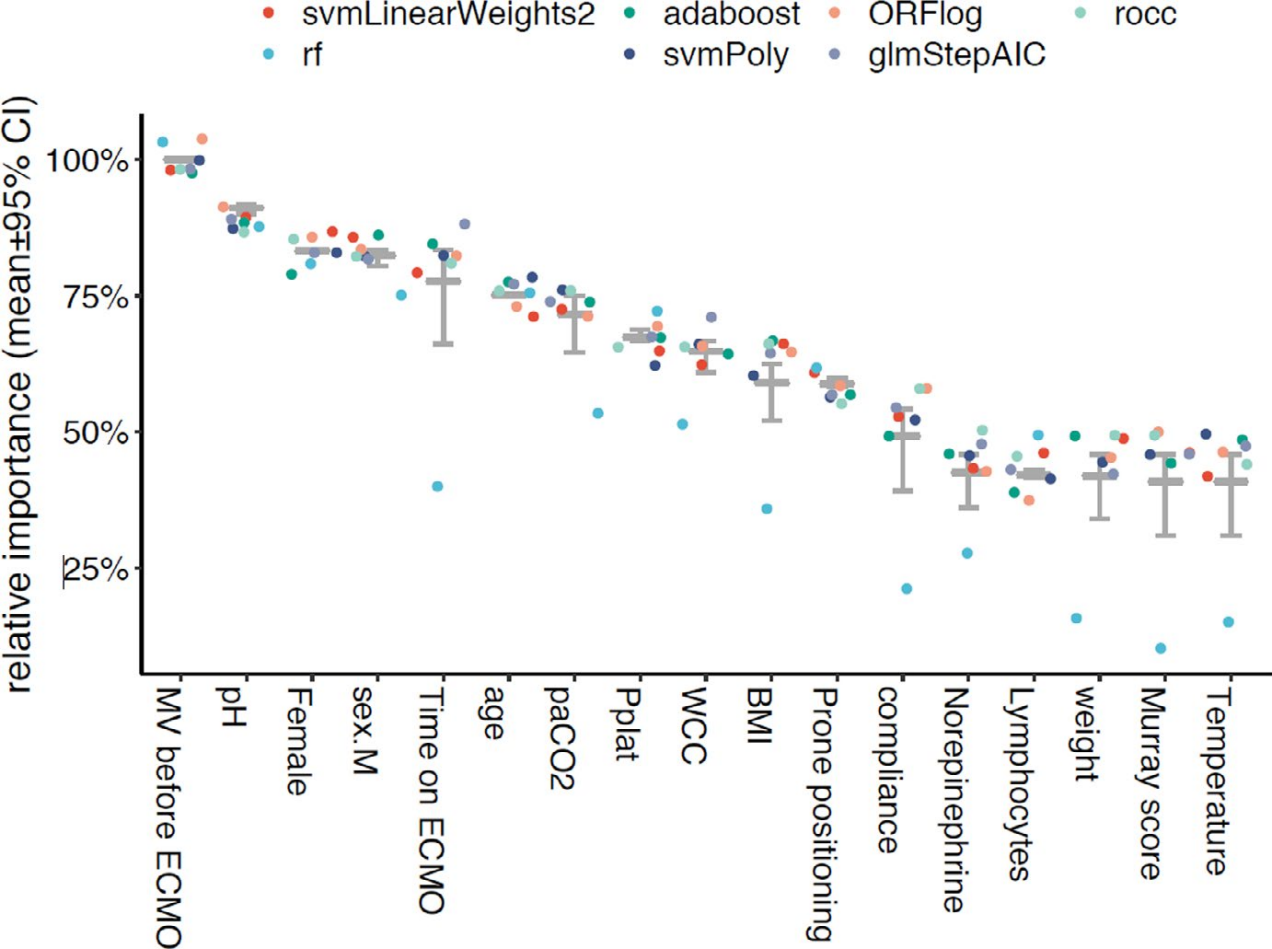
Machine learning graph with predictive model of ICU death

The comparative analysis between survivors and non‐survivors highlights that survivors had a significantly higher pH before the implementation of ECMO (7.48 ± 0.03 vs. 7.32 ± 0.14, *p* = 0.019), a shorter mechanical ventilation duration before ECMO (91 ± 58 h vs. 208 ± 34 h, *p* = 0.01), a shorter time under ECMO (246 ± 102 days vs. 588 ± 294 days, *p* = 0.038), and a shorter ICU length of stay (17 ± 6 days vs. 32 ± 12 days, *p* = 0.016). Therefore, all the patients that had received beyond 7 days of mechanical ventilation before applying ECMO ultimately died (Figure 5). No other parameter before ECMO implantation appeared significantly different between these two groups. The parameters between survivors and non‐survivors are presented in Table 4. Using machine learning to create a predictive model of ICU death, the most significant predictive variable was mechanical ventilation time (hours) before ECMO implantation (Figure 6).

**TABLE 4:** Demographic, biological, ventilatory, hemodynamic, and therapeutic parameters in survivors and non‐survivors of the patients implanted with VV‐ECMO

	Survivors	Non‐survivors	*p*
Gender (F/M)	4 F/0 M	1 F/5 M	0.01
Age (years)	57 ± 4	63 ± 5	NS
Weight (kg)	82 ± 7	75 ± 8	NS
BMI (kg/m^2^)	31 ± 5	26 ± 3	NS
SAPS II	56 ± 3	60 ± 11	NS
CRP (mg/l)	184 ± 106	216 ± 72	NS
Procalcitonine (µg/l)	0.59 ± 0.38	0.98 ± 1.25	NS
WCC (G/l)	5.8 ± 1.4	10.8 ± 6.5	NS
Lymphocytes (G/l)	0.51 ± 0.31	0.64 ± 0.1	NS
N/L ratio	20 ± 21	17 ± 11	NS
D‐dimers (ng/ml)	2787 ± 1826	6917 ± 4622	NS
Us‐Troponine (ng/l)	42 ± 29	142 ± 290	NS
Lactate (mmol/l)	1.3 ± 0.3	2.1 ± 1.5	NS
Creatinine (µmol/l)	68 ± 13	69 ± 32	NS
Urea (mmol/l)	7.6 ± 1.6	9.0 ± 4.1	NS
CVVHDF	0	0	NS
Temperature (°C)	36.8 ± 0.8	37.5 ± 0.8	NS
HR (BPM)	83 ± 20	84 ± 12	NS
MAP (mmHg)	79 ± 13	72 ± 4	NS
Ventilatory mode	ACV	ACV	NS
P/F (kPa)	10 ± 2	10 ± 3	NS
Vt (ml)	409 ± 33	359 ± 62	NS
Vt/PBW (ml/kg)	5.7 ± 0.7	5.6 ± 0.8	NS
PEEP (cmH_2_O)	11 ± 1	12 ± 2	NS
RR (/min)	21 ± 2	23 ± 3	NS
Crs (ml/cmH_2_O)	21 ± 1	17 ± 5	NS
pH	7.48 ± 0.03	7.32 ± 0.14	0.019
PaCO_2_ (kPa)	5.4 ± 1.3	8.1 ± 1.3	NS
Bicarbonate (mmol/l)	32.9 ± 8.1	30 ± 4.3	NS
Base Excess (mmol/l)	6.7 ± 5.8	6.6 ± 6.3	NS
Hb (g/l)	109 ± 29	99 ± 8	NS
Thrombocytes (G/l)	269 ± 101	376 ± 115	NS
NMB (%)	100%	100%	NS
NOR (µg/kg/min)	0.04 ± 0.04	0.11 ± 0.11	NS
Prone postioning (nb)	1.5 ± 0.6	2.7 ± 1.1	NS
iNO (%)	100	100	NS
iNO flow (ppm)	12.5 ± 2.9	12.5 ± 4.5	NS
AntiXa before ECMO (UI/ml)	0.22 ± 0.2	0.28 ± 0.04	NS
AntiXa under ECMO (UI/ml)	0.28 ± 0.04	0.31 ± 0.07	NS
Mechanical ventilation (days)	4 ± 2	9 ± 2	0.024
Tracheotomy (%)	20	66	NS
MURRAY score	3.4 ± 0.1	3.5 ± 0.2	NS
Time on ECMO (days)	9 ± 3	24 ± 12	0.024
ICU length of stay (days)	17 ± 6	32 ± 12	0.016
Hospital length of stay (days)	35 ± 11	35 ± 12	NS

NS (non‐significant) indicates no significant statistical difference between survivors and non‐survivors.

BMI, body mass index; CRP, C‐reactive protein; Crs, compliance of respiratory system; cvvhdf, continuous Veno‐venous HemoDiaFiltration; Hb, hemoglobin; HR, heart rate; iNO, inhaled nitric oxide; MAP, mean arterial pressure; n/l, neutrophils/lymphocytes ratio; NMB, neuromuscular blockers; NOR, norepinephrine; P/F, PaO_2_/FiO_2_ ratio; PaCO_2_, arterial carbon dioxide partial pressure; PBW, predicted body weight; PEEP, positive end‐expiratory pressure; RR, respiratory rate; Vt, tidal volume; WCC, white cells count.

## 4 DISCUSSION

The main finding of this single‐center, retrospective cohort study in patients with severe ARDS due to COVID‐19 pneumonia initiated on VV‐ECMO was that all survivors benefited from early ECMO implementation in both univariable‐ and machine learning‐based multivariable analyses. The patients who were implanted beyond 7 days of mechanical ventilation all died. This study also confirms that COVID‐19 pneumonia causing severe ARDS requiring VV‐ECMO is a serious pathology with a high mortality rate (60% in this cohort) in comparison to the mortality of classical ARDS (Combes et al., 2018; Peek et al., 2009).

Current knowledge on ECMO use in COVID‐19 infection remains limited to small series and registry data. Indeed, although it can meet the ARDS Berlin definition (Force et al., 2012), COVID‐19 pneumonia is a specific disease with specific phenotypes. Its main characteristic is the dissociation between the severity of hypoxemia and maintenance of relatively good respiratory mechanics. We described this phenomenon when the first COVID‐19 patients were admitted to our ICU (Bendjelid & Raphael, 2020). During the first days of mechanical ventilation, some patients showed good pulmonary compliance with preserved pulmonary mechanics (Bendjelid & Raphael, 2020). It appears that at the initial stage, SARS‐CoV‐2 pneumonia induces pulmonary edema of the high permeability type with a significant capillary leak syndrome and a significant venous mixture concomitant with a loss of hypoxic vasoconstriction (Bendjelid & Raphael, 2020).

However, some patients exhibit a typical inflammatory ARDS phenotype with large consolidations predominant in the dependent lower lobes. The current condition generates large areas of true pulmonary shunting (unventilated but perfused lung regions), which further worsens oxygenation, decreases respiratory compliance, and may require higher PEEP, lower tidal volume, prone positioning, neuromuscular blocking agents, and in the worst case, VV‐ECMO (Bendjelid & Raphael, 2020). In this regard, two types of patients have been proposed by Gattinoni et al. (“non‐ARDS”, type 1, and ARDS, type 2) with different pathophysiology (Gattinoni, Chiumello, Caironi, et al., 2020). In 20–30% of these COVID‐19 patients admitted to the intensive care unit (ICU), severe hypoxemia was associated with low compliance values (< 40 ml/cmH_2_O), indicating severe ARDS. It is certainly possible that their lower compliance (i.e., lower gas volume and decreased recruitability) was due to the natural course of the disease, but we cannot exclude the possibility that this severity of damage (increased edema) results, in part, from initial breathing management and self‐induced lung injury (P‐SILI) syndrome (Marini & Gattinoni, 2020). All patients who received ECMO seem to belong to this second ARDS phenotype due to the low pulmonary compliances measured before ECMO implantation.

The use of ECMO in severe ARDS has long been debated over the past 10 years. Many hopes have been founded on the ECMO to Rescue Lung Injury in Severe ARDS (EOLIA) study, comparing early ECMO versus conventional mechanical ventilation (Combes et al., 2018). Unfortunately, the study was stopped for reasons of futility (failure to demonstrate a difference in 60‐day mortality of 20%) and failed to show a significant improvement in mortality (35% vs. 46%; relative risk [RR] 0.76; 95% confidence interval [CI] 0.55–1.04, *p* = 0.09). However, a recent literature review and a meta‐analysis involving two randomized controlled trials and three observational studies, with a total of 1055 patients, showed that VV‐ECMO in severe ARDS allowed for a significant reduction in 60‐day mortality compared to that of conventional mechanical ventilation (Munshi et al., 2019). A study of 83 COVID‐19 patients with ARDS placed on ECMO showed that the pre‐ECMO characteristics of patients with COVID‐19 indicated great severity of ARDS before ECMO implantation (Schmidt et al., 2020). Their PaO_2_/FiO_2_ ratio in particular (62 ± 18 mmHg) was lower than that of the patients included in the EOLIA trial (73 ± 30 mmHg) while pre‐ECMO driving pressure, respiratory system compliance, mechanical power and other respiratory and ventilatory and ventilator parameters were similar in both studies. In addition, 94% of patients in this study benefited from prone positioning before the implementation of ECMO compared to 56% in the EOLIA study and 81% of patients benefited from prone positioning under ECMO compared to 10% in the EOLIA trial. Moreover, patients with COVID‐19 stayed longer on ECMO (20 days, IQR 10–40 vs. 11 days, IQR 7–18 in the EOLIA trial) and longer in the ICU (36 days, IQR 23–60 vs. 23 days, IQR 13–34 in the EOLIA study). Furthermore, the survival of ECMO‐rescued patients with COVID‐19 was similar to that reported in the EOLIA trial. Finally, antibiotic‐treated ventilator‐associated pneumonia rate was higher (87%) in COVID‐19 patients than for patients in the EOLIA trial (39%). This might indicate the longer period under mechanical ventilation or specific SARS‐CoV‐2 induced immunodeficiency.

The evolution of COVID‐19 appears to include an important endothelial insult with histological evidence of endothelial dysfunction (Pons et al., 2020) that also mandate a vascular and a rheological approach (Marini & Gattinoni, 2020). Coagulopathies of various etiologies have been described in COVID‐19 patients (Iba et al., 2020), with an increased risk of venous thromboembolism (VTE) (Trigonis et al., 2020) and elevated D‐dimer values (Zhou et al., 2020) predicting poorer outcomes (Tang et al., 2020). In the present study, there was no significant difference between survivors and non‐survivors regarding the levels of D‐dimer. In a case series on 51 COVID‐19 patients, 8 presented with massive PE, while 4 developed PE while on VV‐ECMO (Hekimian et al., 2020), a condition which was not reported either in the EOLIA trial or in the international multicenter prospectus LIFEGARDS cohort (Combes et al., 2018; Schmidt et al., 2019). In this study, no patient developed PE, despite having rather high D‐dimer levels. This finding is probably due to an early identification of these VTE phenomena and higher anticoagulation levels that have been applied to our patients.

In the first single‐center, retrospective, observational study enrolling 52 critically ill adult patients with SARS‐CoV‐2 pneumonia admitted to the ICU of Wuhan Hospital, 5 (83%) of six patients receiving ECMO died (Yang et al., 2020). In a letter, Brandon M. Henry raised concerns about the potential harm of ECMO therapy for COVID‐19 with respect to the lymphocyte count (Henry, 2020). Our results show a transient drop in the lymphocyte count the day after ECMO insertion, with a return to normal values at day 3. This result is consistent with the results published in a case series of 12 ECMO COVID‐19 patients showing the same results (Huette et al., 2020). However, the authors found that most severe COVID‐19 cases had persistently low lymphocyte counts, which our study failed to show.

In a North American series of 6 COVID‐19 patients with ARDS who were placed on ECMO, a patient died of intracranial hemorrhage (ICF) (Osho et al., 2020). In a cohort study of 3824 COVID‐19 patients, neuroimaging was done in 755 patients, with 37 having radiographic evidence of ICF (Dogra et al., 2020). In our series, the patient with ICF had an intermediate level of anticoagulation. The mechanisms responsible for extrapulmonary damage in COVID‐19, in particular ICF, remain unclear. Pragmatism prompts us to consider an adequate level of anticoagulation for these patients on ECMO. Indeed, the presence of microangiopathies and microthrombi can also predispose the patient to microinfarcts within multiple organs, further exacerbating the state of multiorgan injury and failure.

Our study has some limitations. First, our analyses are retrospective and limited to the recorded data. Nevertheless, there are no missing data among the collected variables. Second, as this was a single‐center study, we cannot externally validate our results. Third, due to its small size, the results of statistical analyses should be interpreted with caution. Larger cohort studies should make it possible to clarify certain important aspects, such as the timing of implementation of ECMO during the evolution of the respiratory illness of these patients. Despite these limitations, this study is, to our knowledge, the first to show that a duration of pre‐ECMO positive‐pressure ventilation greater than 150 h seems to be predictive of mortality. Moreover, using a machine learning algorithm, we were able to fit predictive multivariable models that confirm the key role of mechanical ventilation time before ECMO implantation.

## 5 CONCLUSION

The present retrospective single‐center study is one of the first case series describing VV‐ECMO outcomes in COVID‐19 patients. Our results suggest that VV‐ECMO can be safely utilized in appropriately selected COVID‐19 patients with refractory hypoxemia. The main information that can be deduced from this study is that VV‐ECMO must be considered early in the management of patients with severe ARDS. Late ECMO therapy (i.e., beyond the seventh day of mechanical ventilation) seems futile.

## CONFLICT OF INTEREST

The authors declare that they have no competing interests.

## AUTHORS’ CONTRIBUTIONS

RG, BA, AMC, DDC, MEB, MM and TF collected and interpreted the data. RG, DL, and KB analyzed the patient data. RG, DL, BA, ADC, and KB wrote the manuscript. All authors read and approved the final manuscript.

## ETHICAL APPROVAL

The local ethics committee approved the study and waved the informed consent (BASEC number: 2020–00917).

## Data Availability

The datasets used and/or analysed during the current study are available from the corresponding author on reasonable request.
